# Parkinson’s tremor

**DOI:** 10.1186/2050-5736-3-S1-O2

**Published:** 2015-06-30

**Authors:** Jeff Elias, Robert Dallapiazza

**Affiliations:** 1 University of Virginia, Charlottesville, VA, United States

## Background/introduction

Tremor associated with Parkinson’s disease can be medication resistant and disabling. A clinical trial of stereotactic focused ultrasound thalamotomy is underway.

## Methods

This pilot study is being conducted in two centers with a randomized, controlled study design using blinded and validated rater assessments and a sham procedure. The primary outcome variable is hand tremor and the Unified Parkinson’s disease rating scale in the medicated state at three months. Comprehensive cognitive assessments before and after the procedure are being conducted for additional safety. A total of thirty patients will be enrolled.

